# Editorial: Advances in embedded systems and signal processing in auditory and audiological research

**DOI:** 10.3389/fnins.2026.1809859

**Published:** 2026-03-10

**Authors:** Tamas Harczos, Stefan Zirn

**Affiliations:** 1 Department of Applied Computer Science, University of Applied Sciences Erfurt, Erfurt, Germany; 2 Department of Electrical Engineering, Medical Engineering and Computer Science, Hochschule Offenburg, Offenburg, Germany

**Keywords:** auditory model, auditory modeling, basilar membrane, cochlea, cochlear implant, hearing, hearing aid, signal processing

Over the last decade, auditory and audiological research has been shaped by rapid progress in embedded systems, signal processing, and machine learning, alongside more exploratory developments in neuromorphic hardware. Compact, low-power devices now support signal processing chains that begin at the sensor level and extend to closed-loop interaction with neural tissue. Yet, several long-standing challenges remain unresolved: robust speech understanding in complex acoustic scenes, faithful neural coding with electrical or optical implants, and truly transparent virtual or augmented auditory environments.

This Research Topic brings together four contributions that exemplify how embedded systems and signal processing can move us toward these goals. They span bio-inspired frontends, real-time objective measures for cochlear implants (CIs), binaural strategies modeled on efferent auditory control, and synchronized bilateral processors that can deliver explicit interaural timing cues. Across very different platforms and levels of abstraction, all four papers share three principles: (i) inspiration from auditory neuroscience, (ii) implementation on realistic or deployable embedded hardware, and (iii) a clear line of sight to clinical or technological application.

## Bio-inspired neuromorphic sensing and efferent feedback

Durstewitz et al. couple an adaptive microelectromechanical systems (MEMS)-based “cochlea” to a model of the lateral superior olive (LSO) and medial nucleus of the trapezoid body (MNTB) to study sound-source localization based on interaural level differences (ILDs). Their MEMS cantilevers behave as frequency-selective artificial hair cells whose gain and bandwidth can be tuned via thermo-mechanical feedback. The LSO-MNTB stage is implemented as a conductance-based, neuromorphic network that can run on dedicated hardware.

The central question is how efferent-like feedback from the LSO back to the sensors can improve ILD coding. Using a combination of measured MEMS responses and simulations, the authors show that appropriately configured feedback can (i) compensate left-right mismatches caused by fabrication tolerances, (ii) shift and reshape ILD tuning curves, and (iii) induce rich temporal dynamics where onset responses and time-to-peak carry additional spatial information. Importantly, these effects arised in a closed sensor-processor loop that was amenable to embedded implementation. This work demonstrates, in a concrete hardware-compatible architecture, how efferent control can both robustify a bio-inspired frontend and increase the flexibility of spatial coding.

## Fast, embedded objective measures for cochlear implants

In clinical CI fitting, electrically evoked compound action potentials (ECAPs) are a valuable objective measure, but conventional forward-masking paradigms are slow because each raw waveform must be transmitted from the implant to an external computer and averaged offline. Yang et al. introduce “FastCAP” for the Nurotron CI system, a paradigm that moves the averaging into the implant hardware. Multiple recordings are accumulated on-chip and only the averaged waveform is transmitted, reducing telemetry demands.

Compared to the standard ECAP procedure, FastCAP reduced the measurement time from approximately 340–47 s for the same set of electrodes and levels, while preserving N1 amplitude and latency, and showing high test-retest reliability. Thresholds derived from FastCAP correlated significantly with behavioral T- and C-levels in a considerable subset of participants, and the across-electrode profiles often resembled behavioral profiles when appropriate metrics were used. The work is a model example of how re-thinking data flow and exploiting on-device computation can turn an established but time-consuming protocol into a clinically practical tool. It also illustrates a more general design principle for embedded neuroprosthetic systems: use scarce telemetry bandwidth for distilled, task-relevant summaries rather than raw data.

## Binaural frontend processing inspired by the medial olivocochlear reflex

Earlier work from Lopez-Poveda and colleagues showed that a binaural backend strategy (“MOC1”) that dynamically modulates CI compression based on contralateral activity -analogous to the medial olivocochlear reflex (MOCR)- can improve speech-in-noise performance. However, MOC1 requires one-to-one pairing of channels and identical mapping on both sides, which limits its applicability in real clinical maps.

In this contribution to the special topic, Lopez-Poveda et al. present a frontend implementation (“MOCFE”) that operates entirely in the acoustic domain, preceding MED-EL's FS4 CI strategy. Using frame-based spectral expansion controlled by time-weighted contralateral levels, MOCFE approximates the functional effect of contralateral gain reduction that MOC1 achieves at the electrical backend, but without imposing constraints on electrode configuration or mapping.

In bilateral MED-EL users, MOCFE provided speech-reception-threshold (SRT) benefits in fluctuating noise that were comparable to the backend MOC strategy for most spatial configurations, particularly when target and masker were spatially separated. Localization performance in quiet and in noise were slightly improved -or at least not degraded- by both binaural MOC strategies relative to standard FS4. Conceptually, this work shows that MOCR-like control can be moved to a purely acoustic frontend, which greatly facilitates integration into existing clinical devices.

## Real-time bilateral synchronization and explicit ITD coding

Bilateral CI (BiCI) users typically localize sound largely based on ILDs, in part because current clinical processors are unsynchronized and provide poor access to interaural time differences (ITDs), especially at high stimulation rates. Using the portable CCi-MOBILE platform, Borjigin et al. implement synchronized bilateral processing with mixed-rate strategies that explicitly encode ITDs in selected low-rate channels while maintaining high-rate stimulation on the remaining CI channels to convey sufficient information for speech understanding.

Acoustic recordings from behind-the-ear microphones show that, for the same free-field stimuli, the experimental strategies transmit ITDs to the electrical domain with greatly improved fidelity compared to unsynchronized clinical ACE processing, while ILDs remain similar across strategies. One mixed-rate strategy personalizes the low-rate channel to the electrode pair with the best ITD sensitivity as determined in direct-stimulation tests.

Despite this, in acute horizontal localization tests, BiCI listeners did not show consistent improvements over clinical processors. The lack of improvement in localization performance was probably because ILDs continued to be the dominant cue in acute localization testing. The study therefore delivers two important messages: Technically, synchronized portable processors like CCi-MOBILE can deliver high-quality ITD cues in realistic listening conditions. Perceptually, simply making ITDs available may not suffice; listeners may need extended experience or training, and perhaps modified stimulus statistics, to integrate ITDs into their spatial perception.

## Outlook

Taken together, the contributions in this Research Topic illustrate how embedded systems and signal processing are reshaping auditory research and neuroprosthetic design:

- Bio-inspired, adaptive frontends tightly couple physical transducers to neural models and efferent-like control, enabling robust, energy-efficient sensing and flexible spatial coding.
- Moving computation and averaging into implanted hardware can transform slow, research-grade measurements into clinically viable tools.
- Binaural strategies that emulate brainstem efferent mechanisms can now be placed either at the electrical backend or at a processor-agnostic acoustic frontend, opening deployment paths across heterogeneous clinical maps and devices.
- Synchronized, portable research processors bridge the gap between highly controlled direct-stimulation studies and complex real-world environments, and will be essential for long-term “take-home” evaluations of novel strategies.

Future work will likely combine these directions with modern AI and machine learning methods. For example, using learned models of human hearing to guide sensor adaptation, personalize binaural processing, or predict when and how to engage efferent-like control. As the field advances, an important challenge will be to maintain a strong bidirectional link between physiological knowledge and embedded implementations: using neuroscience to inspire algorithms and hardware, and using embedded systems to test, refine, and sometimes challenge our understanding of auditory processing in realistic conditions.

